# Effect of endometrial biopsy method on ribonucleic acid quality and gene expression analysis in patients with leiomyoma

**DOI:** 10.1016/j.xfre.2023.11.006

**Published:** 2023-11-14

**Authors:** Thea Falkenberg Mikkelsen, Maria Vera-Rodriguez, Gareth Greggains, Péter Fedorcsák, Kirsten Hald

**Affiliations:** a Department of Gynecology, Oslo University Hospital, Oslo, Norway; b Department of Reproductive Medicine, Oslo University Hospital, Oslo, Norway; c Department of Microbiology, Oslo University Hospital, Oslo, Norway; d Institute of Clinical Medicine, University of Oslo, Oslo, Norway

**Keywords:** Pipelle, endometrial biopsy, low-pressure suction curette, resectoscope loop

## Abstract

**Objective:**

To compare ribonucleic acid (RNA) quantity and purity in tissue collected with different endometrial sampling methods to establish the optimal tool for use in endometrial gene expression studies.

**Design:**

Observational study.

**Setting:**

University hospital.

**Patients:**

Fourteen patients with submucosal leiomyomas.

**Interventions:**

Unguided biopsies were obtained using a low-pressure suction device before hysteroscopy from 14 patients with submucosal leiomyomas followed by guided biopsy with a resectoscope loop. Fifty-seven samples were collected: 25 obtained using a suction device and 32 with a loop.

**Main Outcome Measures:**

Total biopsy weight, RNA purity, and RNA yield for each collection method. After complementary deoxyribonucleic acid synthesis, *HOXA10* expression was measured by quantitative polymerase chain reaction in the endometrium overlying and remote from the leiomyoma, as similar expression throughout the cavity was a prerequisite for the use of unguided biopsy method.

**Results:**

The median weight of the samples was significantly larger when obtained with the low-pressure suction device than with the resectoscope loop (153 vs. 20 mg). The RNA yield was similar (suction curette, 1,625 ng/mg; resectoscope loop, 1,779 ng/mg). The A260-to-A280 ratio was satisfactory for 94.7 % of the samples, with no difference between the groups. The endometrial expression of *HOXA10* was similar in areas overlying the leiomyoma compared with that in remote endometrial sites (2^−ΔCt^ = 0.0224 vs. 0.0225).

**Conclusions:**

Low-pressure endometrial suction devices provide tissue samples with acceptable RNA purity and quantity for gene expression studies. The expression of *HOXA10* did not differ between endometrial sampling sites even in the presence of leiomyomas.

Uterine leiomyomas are tumors originating from the myometrium. Although leiomyomas are benign, they can cause abnormal bleeding, dysmenorrhea, pelvic pressure, and reduced fertility (1, 2, 3, 4). Uterine leiomyomas are often classified according to their location in the uterus: submucosal leiomyomas grow into the uterine cavity, intramural leiomyomas are located within the muscular wall of the uterus, and subserosal leiomyomas grow towards the outside of the uterus (5).

Decreased endometrial receptivity is one of many mechanisms that has been proposed as the pathway through which leiomyomas impair fertility (6, 7). Endometrial receptivity is defined as the ability of the endometrium to successfully attach and nourish the blastocyst (8) and is a strictly regulated process involving multiple molecular pathways (9). Transcriptomic profiling of the human endometrium (10, 11) has uncovered several putative molecular markers of a receptive endometrium, such as the homeobox (HOX) genes *HOXA10* and *HOXA11* (12, 13, 14, 15, 16, 17, 18, 19), which may be altered in endometriosis and uterine diseases (20). Indeed, *HOXA10* is an essential transcription factor for embryo implantation (21, 22), which is globally expressed throughout the uterine glands and stroma (23). The expression of *HOXA10* in the endometrial stromal cells appears to be constant throughout the menstrual cycle, whereas the expression in the epithelial glands is cycle dependent, with a higher expression in the secretory phase (24). The mechanisms through which *HOXA10* executes its role in the implantation process are largely unknown, although several target genes have been identified (25, 26). Recently, 1,830 direct and indirect target genes of *HOXA10*, with a wide variety of functions, were identified through ribonucleic acid (RNA) interference and RNA sequencing analysis of cultured endometrial stromal cells. Of the 10 most differentially expressed genes, at least one (*NDRG2*) has a function in the decidualization process (27). Additionally, the endometrial expression of *HOXA10* has been found to be decreased in women with leiomyomas compared with that in controls in most (14, 15, 19), but not all (16, 18), studies.

Reliability of endometrial gene expression analysis depends on the purity and quantity of extracted RNA (28), necessitating optimization of tissue sampling methods. Low-pressure endometrial suction devices, such as Pipelle (Laboratoire CCD, Paris, France) and Endocell, are commonly used to collect unguided samples for studying endometrial gene expression, also in patients with leiomyomas (14, 16, 17, 18, 19). The main reason for the extended use of low-pressure devices is that these have been shown to be safe, easy to use, and time- and cost-effective (29, 30, 31). An alternative sampling approach is guided biopsy during hysteroscopy, which allows directing the biopsy toward a specific location in the uterine cavity.

Although endometrial suction devices have been shown to provide samples with satisfactory RNA purity (32), generalizability of gene expression data from samples obtained by unguided biopsy remains to be ascertained. Homogeneity in gene expression throughout the uterine cavity is a prerequisite for the use of such devices in gene expression analysis because the exact sampling location in relation to focal features, such as leiomyomas, will not be known when using a suction curette. One previous study on women with submucosal leiomyomas showed similarly low expression levels of *HOXA10* in the endometrium located directly over the leiomyoma and in the endometrium elsewhere in the cavity (15), suggesting that leiomyomas have a global effect on the endometrial transcriptomic profile.

In this study, we aimed to compare endometrial samples obtained from patients with submucosal leiomyomas using guided and unguided biopsies to determine the optimal tool for use in endometrial gene expression studies. To elucidate possible differences between the sampling methods, we compared purity and quantity of the extracted RNA between collection methods. In addition, we investigated the expression of *HOXA10* in different locations of the uterine cavity using guided biopsies to evaluate spatially distinct effects of leiomyomas on endometrial gene expression.

## Materials and methods

### Study Population

Fourteen patients with submucosal leiomyomas scheduled for a transcervical myomectomy gave written informed consent and were included in the study between March 2019 and February 2022. Only patients aged 18–40 years with regular menstrual cycles (22–35 days) were included. The exclusion criteria were the use of hormonal treatment, pregnancy during the last 3 months, and ongoing lactation.

### Sample Collection

Immediately before and during hysteroscopy, two unguided biopsies were obtained using a low-pressure suction device (Pipelle), and two guided biopsies were obtained using a resectoscope loop (Olympus WA22503D HF Resection Electrode 24 F, Hamburg, Germany) from each participant. The Pipelle device consists of a hollow plastic tube with an external diameter of 3.1 mm and an internal piston. Negative pressure is created in the tube when withdrawing the piston, thus filling the tube with endometrial tissue through a small opening at the tip (33). The two guided biopsies were obtained from the endometrium overlying the leiomyoma and remote from the leiomyoma during each procedure. Two patients were operated in two sessions because of leiomyomas on both sides of the cavity or because of the size of the leiomyoma, obtaining both Pipelle sample and two guided biopsies both times. Nine of the patients also had samples obtained using a Pipelle 3–6 months after the initial surgery. In total, 57 samples were obtained for further analysis of the main outcome measures: 25 Pipelle biopsies (14 Pipelle biopsies from the initial surgery, 2 Pipelle biopsies from the second surgery, and 9 Pipelle biopsies after 3–6 months) and 32 resectoscope loop biopsies (28 loop biopsies from the initial surgery and 4 biopsies from the second surgery). In addition, 25 Pipelle biopsies were collected for histologic analysis (1 sample during each session). Of the guided biopsies, 28 and 5 were obtained using a cold loop and diathermy, respectively. After collection, the biopsies were rinsed in a sterile tube containing approximately 40 mL of phosphate-buffered saline (product No. D8537; Sigma-Aldrich, St. Louis, MO) and immediately transferred to an RNase-free tube (Sigma-Aldrich) with 2.5-mL RNAlater (Sigma-Aldrich) or put in formalin for histologic assessment of the endometrial stage by the Noyes criteria. The tubes with RNAlater-treated samples were kept overnight at 4°C. On the next day, the samples were removed from the RNAlater with sterile forceps, retubed, and further stored at −80°C.

### RNA Isolation

Samples were thawed at room temperature and weighed on a Sartorius CP225D Analytical Balance (Data Weighing Systems, Inc., Wood Dale, IL). Up to 25 mg of biopsy material was directly used for RNA extraction with an RNeasy Mini Kit (Qiagen, Hilden, Germany). The full procedure was performed according to the manufacturer’s instructions. The extracted RNA was eluted in 80 μL of RNase-free (not diethyl pyrocarbonate–treated) water (product No. AM9937; Thermo Fisher Scientific, Waltham, MA). The RNA quantity and purity were assessed with a NanoDrop Spectrophotometer (Thermo Fisher Scientific), with purity being evaluated according to the A260-to-A280 ratio. As the maximum absorbance of nucleic acids is at 260 nm and that of proteins is at 280 nm, a low A260-to-A280 ratio indicates contamination with proteins and is routinely used to assess RNA purity (34). A260/A280 values between 1.9 and 2.1 indicate pure RNA according to the RNeasy Mini Kit documentation, whereas values up to 2.3 are routinely seen for pure RNA when dissolved in 10-mM Tris, pH 7.5, and lower values are observed when dissolved in pure water (35, 36). Therefore, in this study, the A260/A280 values between 1.9 and 2.2 were considered satisfactory. Samples with A260/A280 values outside the range of 1.9–2.2 were considered low-purity samples. Purified RNA was stored at −80°C until further processing.

### Real-Time Quantitative Polymerase Chain Reaction

Isolated total RNA samples were diluted to 50 ng/μL before complementary deoxyribonucleic acid (cDNA) synthesis by adding RNase-free water. Complementary deoxyribonucleic acid synthesis was performed using SuperScript IV VILO Master Mix with ezDNase (Thermo Fisher Scientific) following the manufacturer’s instructions. Briefly, genomic deoxyribonucleic acid was digested by adding the following to 7 μL of isolated RNA (350 ng): 1 μL of 10X ezDNase Buffer; 1 μL of RiboLock RNase Inhibitor; and 1 μL of ezDNase enzyme. Genomic deoxyribonucleic acid digestion was accomplished by incubating the samples at 37°C for 2 minutes. After a 1-minute incubation in a cold rack, 4 μL of SuperScript IV VILO Master Mix and 6 μL of nuclease-free water were added to each sample. Complementary deoxyribonucleic acid synthesis was achieved by incubating the samples at 25°C for 10 minutes, 50°C for 10 minutes, and 85°C for 5 minutes. Half of the volume of each cDNA sample (10 μL) was diluted 1:10 with RNase-free water for further use in real-time quantitative polymerase chain reaction (qPCR). The remaining cDNA was stored at −80°C.

For gene expression analysis, *HOXA10* was selected as an endometrium-related target gene, in addition to *PPIA* and *YWHAZ* as reference genes on the basis of previous studies (37, 38, 39, 40, 41) and our preliminary investigations showing stable expression of these two genes in patients with leiomyoma (data not shown). Specific primers were designed to span exons and detect all gene isoforms wherever possible (the primer information is listed in Table 1). Quantitative polymerase chain reaction was performed using the PowerTrack SYBR Green Master Mix (Thermo Fisher Scientific). As recommended, the qPCR master mix was prepared by mixing 10 μL of PowerTrack SYBR Green PCR Master Mix 2X, 0.8 μL of 10-μM primer pairs, 5 μL of cDNA, and 4.2 μL of RNase-free water. Amplification of each gene was performed under the following cycling conditions: activation at 95°C for 20 seconds and then 40 cycles of 95°C for 3 seconds followed by 58°C for 30 seconds. Quantitative polymerase chain reaction was performed in an Applied Biosystems ViiA 7 machine (Thermo Fisher Scientific). Each sample was run in triplicate, and positive and negative controls were also included in each plate. For positive controls, 1 μg/μL of Clontech Laboratories Human Universal Reference Total RNA was used. Complementary deoxyribonucleic acid synthesis and qPCR reactions for positive controls were performed as described earlier.

**Table 1:** Primer sequences for gene expression analysis by real-time quantitative polymerase chain reaction.

Target gene	Forward primer	Reverse primer	Product length
*HOXA10*	GGATTCCCTGGGCAATTCCAAA	AGTGTCTGGTGCTTCGTGTAG	99
*YWHAZ*	ACCGTTACTTGGCTGAGGTTGC	CCCAGTCTGATAGGATGTGTTGG	130
*PPIA*	GGCAAATGCTGGACCCAACACA	TGCTGGTCTTGCCATTCCTGGA	161

### Data Analysis

Data with non-normal distribution, including biopsy weight, were compared using the Wilcoxon signed rank test or matched-pairs test, as appropriate. An unpaired or paired *t*-test was used to compare the means for data with a normal distribution, including RNA yield. The χ^2^ test was used to compare the proportion of samples with satisfactory RNA purity between groups. The cycle threshold (Ct) values were obtained using ViiA 7 Software (Thermo Fisher Scientific). Triplicates with a standard deviation of >0.3 were excluded from further analysis. Similarly, samples with technically unreliable data, such us unusual melt curves, were excluded.

The mean Ct values of the reference genes (Ct_ref_) and each gene of interest (Ct_goi_) were calculated. The normalized Ct values for each sample and gene of interest were obtained using the following formula: ΔCt = Ct_goi_ − Ct_ref_. In qPCR, the Ct indicates the fractional cycle number at which the amount of amplified target reaches a fixed threshold; this is true for both the gene of interest (Ct_goi) and control (Ct_ref). As the number of molecules is doubled with each PCR cycle, the relative gene expression X_goi/X_ref can, thus, be calculated from the number of cycles it took to reach the threshold through the following formula: 2^−ΔCt^, where ΔCt = Ct_goi − Ct_ref. In short, the larger the ΔCt, the more doubling cycles it takes to amplify the amount of mRNA molecules of the target gene to the prespecified threshold (42). The ΔCt values were normally distributed, and the paired *t* test was used for comparison.

### Ethical Approval

This study was approved by the Regional Committees for Medical and Health Research (REK, No. 2018/1858 and 66064).

## Results

We collected unguided and guided endometrial biopsies from 14 patients with submucosal leiomyomas. The mean age and body mass index were 37 (range, 27–39) years and 21.6 (range, 18.0–33.1) kg/m^2^, respectively. Of the 14 patients included, 10 were treated for a type 1 leiomyoma, 1 for a type 0 leiomyoma, 1 for a type 2 leiomyoma, 1 for both type 0 and 1 leiomyomas, and 1 for 2 type 1 leiomyomas according to the International Federation of Obstetrics and Gynecology classification (5). A subset of the samples was histologically analyzed for endometrial dating using the Noyes criteria. Seventeen (85%) of 20 analyzed samples were confirmed to be in the secretory stage, with no significant difference between the sample type groups.

### Biopsy Weight

In total, we collected 57 endometrial samples, 25 using a low-pressure suction device (Pipelle) for unguided biopsy and 32 using a resectoscope loop for guided biopsy.

**Figure 1:**
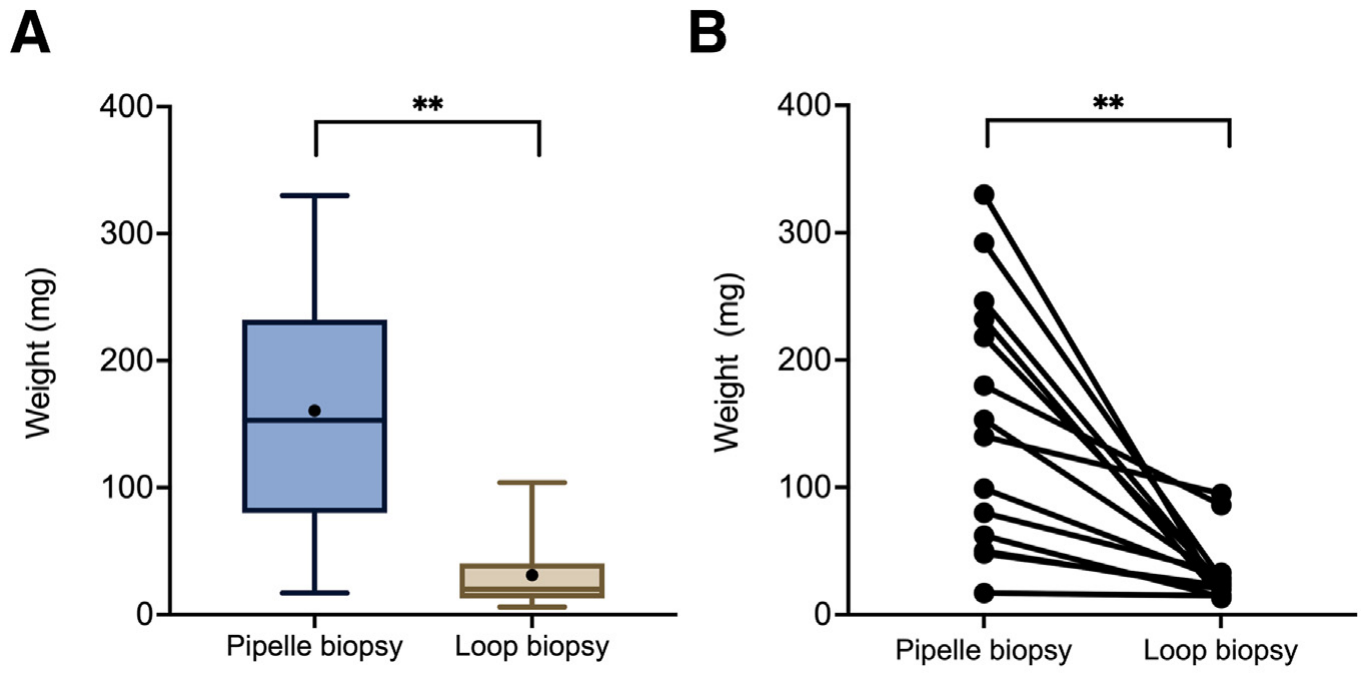
Biopsy weight according to the sampling method. (A) Boxplots of the weight of biopsies collected using the Pipelle and resectoscope loop. The black circle represents the mean value. ∗∗*P*<.001, Wilcoxon signed rank test. (B) A paired chart of biopsy weight between samples obtained using the Pipelle and resectoscope loop in the same patient during the same procedure. The mean weight of the two obtained loop biopsies is reported. ∗∗*P*<.001, Wilcoxon matched-pairs test.

The median weight of the biopsies obtained using the Pipelle device (n = 23; missing data, n = 2) was higher than that from the guided biopsies (n = 30; missing data, n = 2; 153 and 20 mg, respectively; *P*<.01, Wilcoxon signed rank test; Fig. 1A). Because the amount of collected endometrial tissue could be affected by the menstrual cycle phase, paired samples were also compared. In all 14 sample pairs, a lower amount of tissue was collected using guided biopsied vs. the suction device (*P*<.01, Wilcoxon matched-pairs test; Fig. 1B).

### RNA Quantity and Purity

**Figure 2:**
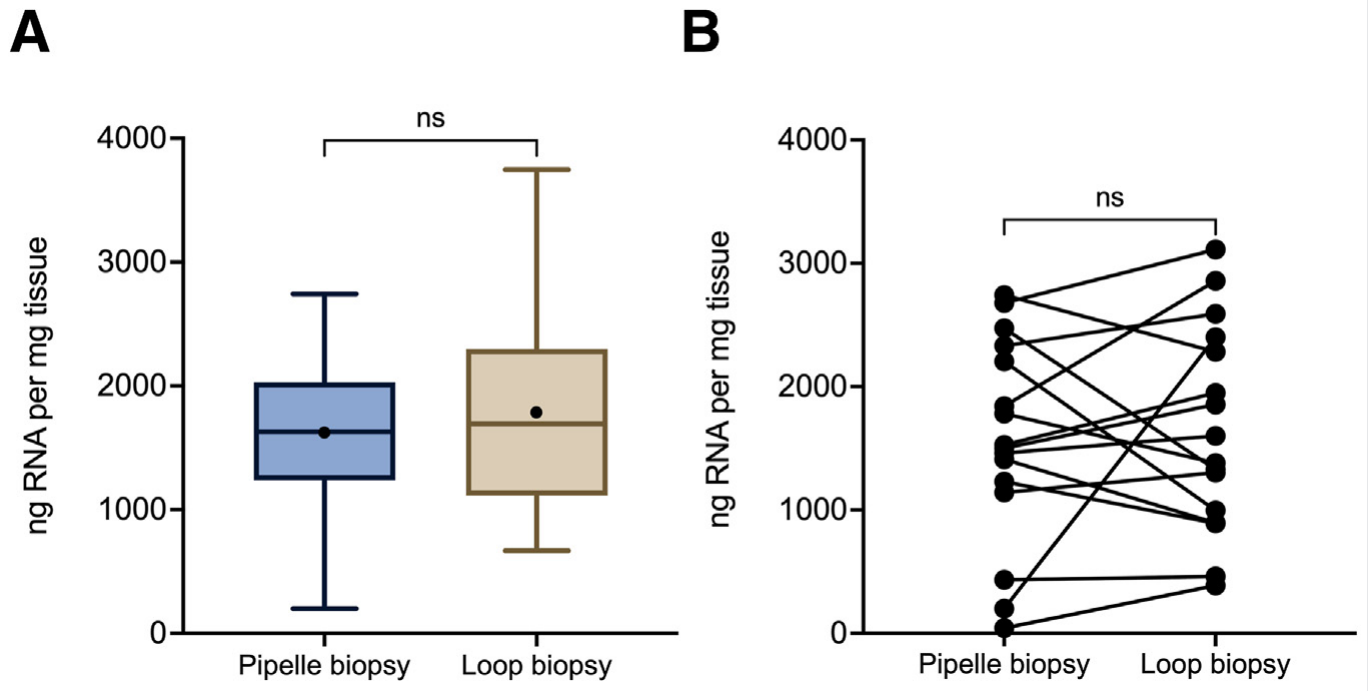
Ribonucleic acid (RNA) yield according to the sampling method. (A) Boxplots of the amount of RNA per milligram of tissue in biopsies collected using the Pipelle and loop. The black circle represents the mean value. ns = nonsignificant, unpaired *t* test. (B) Paired chart of the RNA yield of the samples obtained using the Pipelle and loop during the same procedure. The mean RNA yield of the two obtained loop biopsies is reported. ns = nonsignificant, paired *t*-test.

The mean amounts of RNA obtained per milligram of tissue from Pipelle biopsies (1,625 ng/mg, n = 25) and the loop biopsies (1,779 ng/mg, n = 32) were comparable (*P*=.43, unpaired *t*-test; Fig. 2A). Similarly, paired samples obtained from the same patient (n = 16) had similar RNA yields regardless of whether sampling was with the suction device or resectoscope loop (*P*=.7, paired *t*-test; Fig. 2B).

Ribonucleic acid purity was evaluated by the measurement of the A260-to-A280 ratio. From the 25 samples obtained with the suction device, 23 (92%) showed extracted RNA within the satisfactory range, whereas from the guided biopsies, 31 (97%) of 32 samples yielded RNA of satisfactory purity. No statistically significant differences were found in the purity of the extracted RNA according to the method of biopsy (*P*=.7, χ^2^ test).

### *HOXA10* Expression

**Figure 3:**
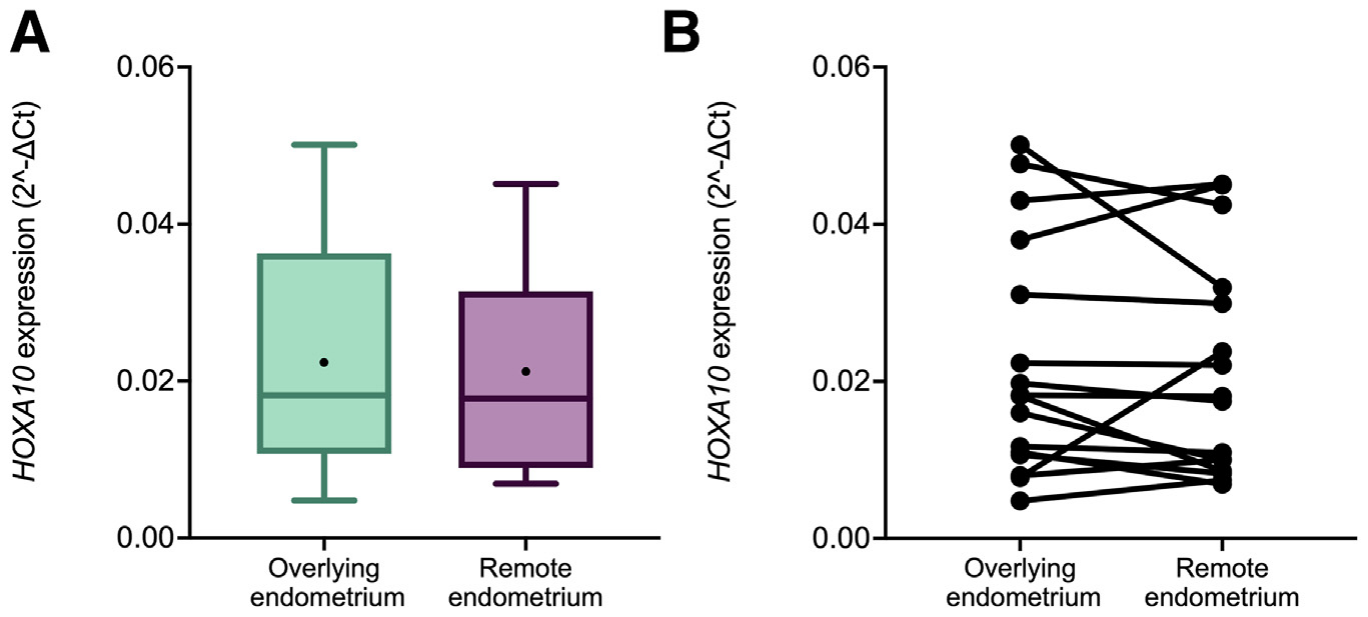
*HOXA10* relative expression according to the biopsy location. (A) Boxplots of the relative *HOXA10* expression levels in all biopsies according to the endometrial location. The black circle represents the mean value. The formula used to transform the cycle threshold (Ct) values to relative gene expression was 2^−ΔCt^. (B) Paired chart of the relative *HOXA10* expression in the endometrium overlying the leiomyoma and that remote from the leiomyoma collected during the same procedure. The formula used to transform Ct values to relative gene expression was 2^−ΔCt^.

To assess spatial differences in the expression of a gene transcript with known relevance for endometrial function in the presence of a localized lesion, such as leiomyoma, the expression of *HOXA10* was evaluated in 30 paired biopsies obtained at different endometrial sites in the same patient: 15 were collected in the area overlying the leiomyoma and the other 15 were from a remote location overlying the normal myometrium. No statistically significant differences in the mean expression of *HOXA10* were found between the biopsies obtained from the overlying area (2^−ΔCt^ = 0.0224) and the paired biopsies from a remote location (2^−ΔCt^ = 0.0225; *P*=.9, paired *t*-test; Fig. 3A). Because of the small sample size, statistical assessment of differences between the *HOXA10* expression of different leiomyoma types could not be performed.

## Discussion

Increasing interest in the effect of leiomyoma on the endometrial transcriptome and markers of endometrial receptivity has created a need for accurate data on the effect of sample collection methods on resultant RNA quantity and quality. Here, we compared endometrial biopsies from an unguided suction device with guided endometrial biopsies, and by evaluating biopsy weight, RNA yield, RNA purity, and *HOXA10* expression, we found suction devices to be superior for sampling because they yielded larger samples with similar RNA purity and quantity.

We chose the widely used Pipelle instrument for unguided biopsies and the resectoscope loop for guided sampling. When comparing Pipelle biopsies with those obtained using the resectoscope loop, we found that Pipelle biopsies provide, on average, six times more tissue. Larger samples may be particularly advantageous for splitting among multiple analyses, for example, combinations of gene expression, transcriptomics, and especially histology. To be able to confirm the menstrual phase by histology is crucial in most endometrial gene expression studies because gene expression varies with the menstrual cycle (43, 44, 45). A minimum biopsy weight needed for histologic dating of endometrial tissue is not defined because few studies include the size or weight of biopsies before preparation. However, a cutoff value of 35 mm^2^ of endometrial tissue surface has been proposed for conclusive diagnosis of malignancies by histologic examination (46). In our experience, it is optimal to view 5–8 mm of tissue in each direction for microscopy, corresponding to a biopsy weight that is preferably exceeding 25–50 mg, depending on thickness. Seventeen (56.7%) of 30 samples of the biopsies in the guided biopsy group had a weight of <25 mg, whereas this was the case for only 1 (4.3 %) of 23 samples in the unguided biopsy group obtained using the Pipelle.

A commonly used alternative to guided endometrial biopsy is hysteroscopic forceps. These are available in sizes from 3 to 10 F (approximately 1–3 mm). To our knowledge, no studies have reported the weight of biopsies obtained with this type of forceps. However, there are studies that include the weight of samples obtained with forceps from the mucosa during gastrointestinal (GI) endoscopy (47, 48). The size of these GI forceps is 1.8–3.4 mm, and these forceps obtain specimens from the mucosa with weights ranging from 3 to 15.5 mg, which are, thus, considerably lower than the weights of samples obtained with the resectoscope loop (range, 6–104 mg, with a median of 20 mg). Although the techniques used for taking biopsies in the GI tract and endometrium are not identical, they are similar, and therefore, we expect that biopsies obtained using the hysteroscopic forceps would also be smaller than those obtained using the resectoscope loop. The use of the resectoscope loop instead of forceps for directed biopsies introduces the possible use of diathermy that could do harm to the tissue. However, in our study, we found that most often, the use of diathermy was unnecessary (27 of 32 samples were obtained without diathermy). It is a limitation of our study that the exact method of obtaining the biopsy with the resectoscope loop was decided by the surgeon. Hence, different techniques had been used, and comparison between the samples obtained with and without diathermy could not be made. Altogether, guided biopsies with the resectoscope loop may yield larger samples than biopsies with forceps but still far smaller than those collected with a Pipelle. Moreover, and importantly, the resectoscope loop is only practical for use in conjunction with an operative hysteroscopic procedure because it requires advanced equipment, blocking of the cervical channel, and a trained surgeon, unlike the low complexity of the Pipelle.

Despite concerns about the accuracy of “blind” endometrial sampling methods (49, 50, 51), Pipelle and other low-pressure suction devices are widely used as a first instrument of choice (29, 52, 53) because it is cost-effective (31) and well tolerated (52, 54) and yields samples of satisfactory quality (55, 56). However, most studies using the Pipelle evaluated suitability for histology and not for gene expression, which requires high RNA quality for robustness of downstream analysis. Unlike the guided biopsies, the Pipelle samples could potentially be contaminated with extraneous, nonendometrial material such as blood and mucus. In this study, we thoroughly rinsed the biopsies in phosphate-buffered saline to avoid this potential bias; however, small remnants of blood could still be present. Nonetheless, our results show that we achieved samples with satisfactory RNA purity at a similar frequency for both methods. Similarly, the amount of RNA obtained per milligram of tissue was similar between methods. In our study, the mean RNA yields were 1,625 ng/mg in the Pipelle biopsies and 1,779 ng/mg in the guided biopsies, which are in line with earlier reports (32).

The endometrium is a heterogeneous tissue composed of a variety of cell types with divergent gene expression profiles (57). Indeed, endometrial epithelial cells display a unique transcriptomic signature with temporal and spatial heterogeneity, dependent on their luminal, glandular, or basal localization (58). However, spatial differences throughout the uterine cavity remain to be described. Nonetheless, intracavitary pathologies may influence the characteristics of the endometrium in a spatial manner (59). The *HOXA10* gene plays a major functional role in the endometrium, and its expression has been shown to be influenced by the presence of leiomyomas (14, 15, 19). Nevertheless, when comparing the endometrium overlying the leiomyoma and that remote from the leiomyoma, we did not find significant differences in *HOXA10* expression, which is in line with a previous study (15). Although our sample size was too small to rule out an absolute difference in the expression levels, no consistent difference was observed for paired data obtained from the endometrium overlying the leiomyoma and that remote from the leiomyoma. These findings suggest that even in the presence of focal pathologies, unguided low-pressure suction devices yield tissue samples that faithfully represent the global gene expression pattern in the endometrium. Consequently, methods that assess progression of the endometrial cycle by transcriptomic analysis, including endometrial receptivity assays, may be robust despite the presence of focal uterine lesions, such as leiomyoma. However, relevant marker genes would require individual evaluation of spatial effects.

## Conclusion

In summary, our results show that Pipelle endometrial sampling is superior to the resectoscope loop method for transcriptomic analysis because it provides a satisfactory amount of material without affecting RNA quality. Moreover, Pipelle sampling is a well-tolerated, cost-effective, and easy method to use compared with the technically challenging and more resource-intensive resectoscope loop biopsy. However, it is important to note that spatially homogeneous gene expression throughout the endometrium is a prerequisite for unguided sampling with a suction device. In cases of spatially non-homogeneous gene expression patterns, guided biopsies with a resectoscope loop would be an appropriate alternative because RNA yields and purity are similar to those obtained with a suction device. However, smaller sample sizes may require multiple biopsies to support additional analyses, particularly histologic examination. In conclusion, our findings confirm that the suction curette method, commonly used in leiomyoma research, is a robust tool for tissue sampling.

## Declaration of Interests

T.F.M. received a training grant from Oslo University Hospital. M.V.-R. has nothing to disclose. G.G. has nothing to disclose. P.F. has nothing to disclose. K.H. has nothing to disclose.
